# The effect of the integration of health services on health care usage among patients with type 2 diabetes in North Karelia, Finland

**DOI:** 10.1186/s12913-021-06059-2

**Published:** 2021-01-13

**Authors:** Katja Wikström, Marja-Leena Lamidi, Päivi Rautiainen, Hilkka Tirkkonen, Petri Kivinen, Tiina Laatikainen

**Affiliations:** 1 Institute of Public Health and Clinical Nutrition, University of Eastern Finland, PO Box 1627, 70211 Kuopio, Finland; 2 Department of Public Health Solutions, Finnish Institute for Health and Welfare, Helsinki, Finland; 3 Joint Municipal Authority for North Karelia Social and Health Services, Joensuu, Finland

**Keywords:** Health services, Integration, Health care, Type 2 diabetes mellitus, Quality of care

## Abstract

**Background:**

The need to improve the care of people with complex care requirements has been driving the reforms integrating care processes. This study examines the effect of the integration of health services on health care usage and the processes and outcomes of care among type 2 diabetes patients.

**Methods:**

Data include all type 2 diabetes patients who lived in North Karelia, Finland, between 2014 and 2018. Health care contacts and glycated haemoglobin (HbA1c) measurements were obtained from the electronic health records. Logistic, Poisson and linear models with generalised estimating equations and the Friedman test were used to study the differences between years.

**Results:**

The health care usage was highest in 2017, the first year of a new organisation, and smallest in the following year. Before the new organisation, the health care usage was lowest in 2014, being slightly higher compared with 2018. Between the last two years, the mean number of contacts per person declined from 3.25 to 2.88 (-0.37, *p* < 0.001). The decreasing pattern seen in total health care usage was most obvious among contacts with primary health care nurses. The number of contacts increased only among specialised care nurses between the last two years. The number of HbA1c measurements was also in its lowest in 2018 but in its highest in 2015. Between the years 2014 and 2018, the difference in the mean number of contacts was − 0.05 (*p* = 0.011) for those not measured, -0.02 (*p* = 0.225) for those measured and within the target level of HbA1c, and 0.12 (*p* = 0.001) for those measured and not at the target level of HbA1c.

**Conclusions:**

Health care integration first increased the health care usage but then brought it to a slightly lower level than before. The changes were most obvious in primary health care nurses’ appointments, and no decline was observed in secondary-level care. Even though the numbers of HbA1c measurements and the proportion measured declined, measurements increased among those with poor glycaemic control. The observed changes might reflect the better targeting and more concordant services in different service units.

## Background

Europe´s population is ageing rapidly. It is also well known that the incidence of chronic diseases – such as cardiovascular diseases, type 2 diabetes and memory diseases – increase with age, as do the co-existence of many chronic diseases (i.e. multimorbidity). Chronic diseases and multimorbidity are often associated with higher mortality, hospitalisations and health care expenditure [1, 2]. The health outcomes of patients with chronic conditions can be remarkably improved, and the complications and costs of care reduced with adequate treatment and the management of risk factors, especially among patients with type 2 diabetes [3, 4]. However, without good coordination and collaborative care, the outcomes of care can be suboptimal.

In particular, the need to improve the care of people with complex care requirements has been driving the reforms integrating care and especially the funding of care [5]. The potential impacts of integrated funding on integrated care are improved access to care, reduced unplanned admissions and readmissions, the reduction of total costs, improved outcomes and quality of care, and improved patient experiences [6]. The experiences and results obtained from service integration have varied. Significantly lower secondary-care use or reduced costs have only been reported on a few occasions [6, 7]. However, improvement in access to care has been observed [6, 8], and it is suggested that integrated care may uncover unmet service needs.

Due to demographic and epidemiological transition, the need to integrate care for people with chronic conditions is widely acknowledged across Europe. In Finland, the main responsibility for organising both health and social services lies with the municipalities (i.e. local authorities), and several governments have attempted reforms aiming to centralise organisational structures in order to improve access to primary care and to integrate services, but the implementation of these reforms are still in progress [9, 10]. According to the recent Health Systems in Transition review [11], Finnish municipalities vary in their ability to deliver health services, and matching service provision to population needs remains a general challenge. In order to organise health and social care services and to respond to the changes in care demand among the ageing population, in some regions smaller municipalities have established joint authorities.

At the beginning of 2017, a major change in the organisation of health services occurred in the eastern part of Finland when the Joint Municipal Authority for North Karelia Social and Health Services (shortly: Siun sote) was established, joining the health service organisations of 14 municipalities. The integration was structural, combining both social and health care responsibilities and including secondary-level care under single management, also integrating finances and resources. The infrastructure of Siun sote service provision included one central hospital and 22 health stations with approximately 400 physicians, 4900 nurses and other health care personnel and 2100 other staff including social workers, fire and rescue workers, assistants, technicians, and administrative personnel. The integration did not have a major effect on infrastructure or amount of staff, but aimed more at new processes in service provision.

In addition, this organisational change was expected to decelerate the increase of the costs of social and health care, to improve work processes between professionals and to meet the complex needs of patients with chronic diseases. Further, it aimed to improve patients´ level of satisfaction with access to care and the continuity of care. Results from previous studies have shown that there is a positive association between patient satisfaction, the continuity and accessibility of care, and treatment outcomes [12–14].

The aim of this study was to analyse the effects of the integration of primary- and secondary-level care on the use of health services and to evaluate the processes and outcomes of care before and after the integration among type 2 diabetes patients.

## Methods

### Study population

Data include all the patients with type 2 diabetes who were living permanently in the North Karelia area between 2014 and 2018. The patients were identified from the regional electronic health records (EHRs) using the ICD-10 code E11. Patients who were diagnosed or died during that time were excluded from that year’s cohort (i.e. only patients with whole-year follow-up were included).

### Variables

Patients’ age, sex, health care contact and glycated haemoglobin (HbA1c) measurements were obtained from the EHRs. All type 2 diabetes–related primary and specialised health care contacts (appointments and phone calls) with nurses, doctors and dentists were included to the analyses. The number of diabetes-related appointments and phone calls were primarily identified using the ICD-10 code E11 and the ICPC-2 code T90. However, the most common multimorbidity-related codes that appeared as the reason for making contact were also taken into account: mental disorders (ICD-10: F20, F25, F31, F32, F33, F34; ICPC-2: P72), cardiovascular diseases (ICD-10: I20–I25, I46, I50, I63-I66 [except I63.6], G45, I70, I79.2; ICPC-2: K78), memory disorders (ICD-10: ​F01–F03, G30; ICPC-2: P20), other follow-up contacts with nurses (ICPC-2: A98, S19, S97, B81) and contacts made for dental care (ICD-10: K02, K04; ICPC-2: D82). As in the recommendations of the Finnish Current Care Guidelines for Diabetes [15], less than 7% (53 mmol/mol) HbA1c was regarded as the target level. All HbA1c measurements were used when counting the annual number of measurements, but only each year’s last HbA1c measurement was used for the target variable.

### Statistical methods

Percentages, means and ranges were used to describe background variables (sex and age), the proportion of health care users and the number of contacts. Logistic, Poisson and linear models with generalised estimating equations (GEEs) were used to study the differences between years in those variables. GEEs account for the correlation structure in data with repeated measurements. Logarithmic transformation was used in a linear model with GEEs for the number [log10(*n* + 0.5)] in order to make the distribution of residuals more symmetric. Also, a non-parametric Friedman test was used in cases where previous models could not be utilised. The Friedman test can only account for those patients who are in the follow-up every year (9553 patients of all 15,277 patients). Percentage points (%p) were used for recording the differences in percentages. The R language and environment for statistical computing (Version 3.5.3) [16] and IBM SPSS Statistics for Windows (Version 25.0) were used in statistical analyses [17]. *P*-values less than 0.05 were regarded as statistically significant and 95% confidence intervals (CIs) were used for statistics.

## Results

The mean age of patients with type 2 diabetes in North Karelia was 68.3 years old in 2014 and it increased to 69.2 years old by 2018 (see Table 1). At the same time, the proportion of women decreased from 46.5–45.6%.

**Table 1:** Background information, health care contacts (appointments and phone calls) and HbA1c measurements

	Annual range	2014	2015	2016	2017	2018	*P*-value
**T2D cohort**							
* N* of T2D patients		11,781	11,967	12,590	12,800	12,969	
Sex, % of women		46.5	46.0	45.8	45.6	45.6	< 0.001^a^
Age, mean	18–117	68.3	68.6	68.7	69.0	69.2	< 0.001^b^
**Health care appointments and phone calls**							
*N* of appointments and phone calls		33,909	36,961	37,367	41,610	37,290	
Proportion of users, %		73.3	74.9	73.5	75.3	72.3	< 0.001^a^
*N* of contacts per person, mean	0-169	2.88	3.09	2.97	3.25	2.88	< 0.001^b^
*N* of appointments per person, mean	0-168	2.54	2.68	2.57	2.75	2.33	< 0.001^b^
*N* of phone calls per person, mean	0–29	0.34	0.41	0.40	0.50	0.55	< 0.001^c^
Proportion of phone calls of all contacts, %		11.9	13.2	13.5	15.3	19.1	< 0.001^a^
**Primary/Specialized care**							
*Primary care*							
*N* of appointments and phone calls		31,224	33,923	34,440	38,296	33,538	
Proportion of users, %		71.6	73.1	71.9	73.7	70.5	< 0.001^a^
*N* of contacts per person, mean	0-167	2.65	2.83	2.74	2.99	2.59	< 0.001^b^
*N* of appointments per person, mean		2.31	2.43	2.33	2.49	2.04	< 0.001^b^
*N * of phone calls per person, mean		0.34	0.41	0.40	0.50	0.55	< 0.001^c^
*Specialised care*							
*N* of appointments and phone calls^+^		2685	3038	2927	3314	3752	
Proportion of users, %		9.4	9.5	9.1	9.5	9.6	0.450^a^
*N* of contacts per person, mean	0-160	0.23	0.25	0.23	0.26	0.29	< 0.001^c^
Proportion of specialized care contacts of all contacts, %		7.9	8.2	7.8	8.0	10.1	< 0.001^a^
**HbA1c measurements**							
*N* of measurements		19,798	19,760	19,423	19,178	17,768	
Proportion of measured, %		76.2	77.0	75.8	76.2	74.9	< 0.001^a^
*N* of measurements per person, mean	0–16	1.41	1.42	1.35	1.36	1.34	< 0.001^d^
HbA1c* < 7%, %		69.7	68.5	69.7	67.3	68.0	< 0.001^a^
*N*of contacts by HbA1c*, mean							
HbA1c not measured		1.14	1.20	1.12	1.21	1.09	0.002^b^
HbA1c < 7%		2.94	3.12	3.09	3.32	2.92	< 0.001^b^
HbA1c > = 7%		4.53	4.83	4.64	5.06	4.65	< 0.001^b^

* The year’s last HbA1c value

^+^ The number of phone calls was 11 in specialised care

^a^Logistic model, ^b^Linear model, ^c^Friedman test, ^d^Poisson model

### Health care usage

Health care usage was highest in 2017, the year when Siun sote started to work, and smallest in the following year 2018; the mean number of all contacts (appointments and phone calls) per person declined from 3.25 to 2.88 (-0.37, *p* < 0.001) (see Table 1). The change was even bigger for appointments, declining from 2.75 to 2.33 (-0.42, *p* < 0.001). Before the new organisation was established, the usage was the lowest in 2014; the mean number of all contacts was at the same level as in 2018 (2.88), but the change in the mean number of appointments was − 0.21 (*p* < 0.001). The mean number of phone calls per person per year increased from 0.34 to 0.55 and phone calls proportion of all forms of contact rose from 11.9–19.1% during the whole follow-up period.

### Primary and specialised care usage

**Fig. 1:**
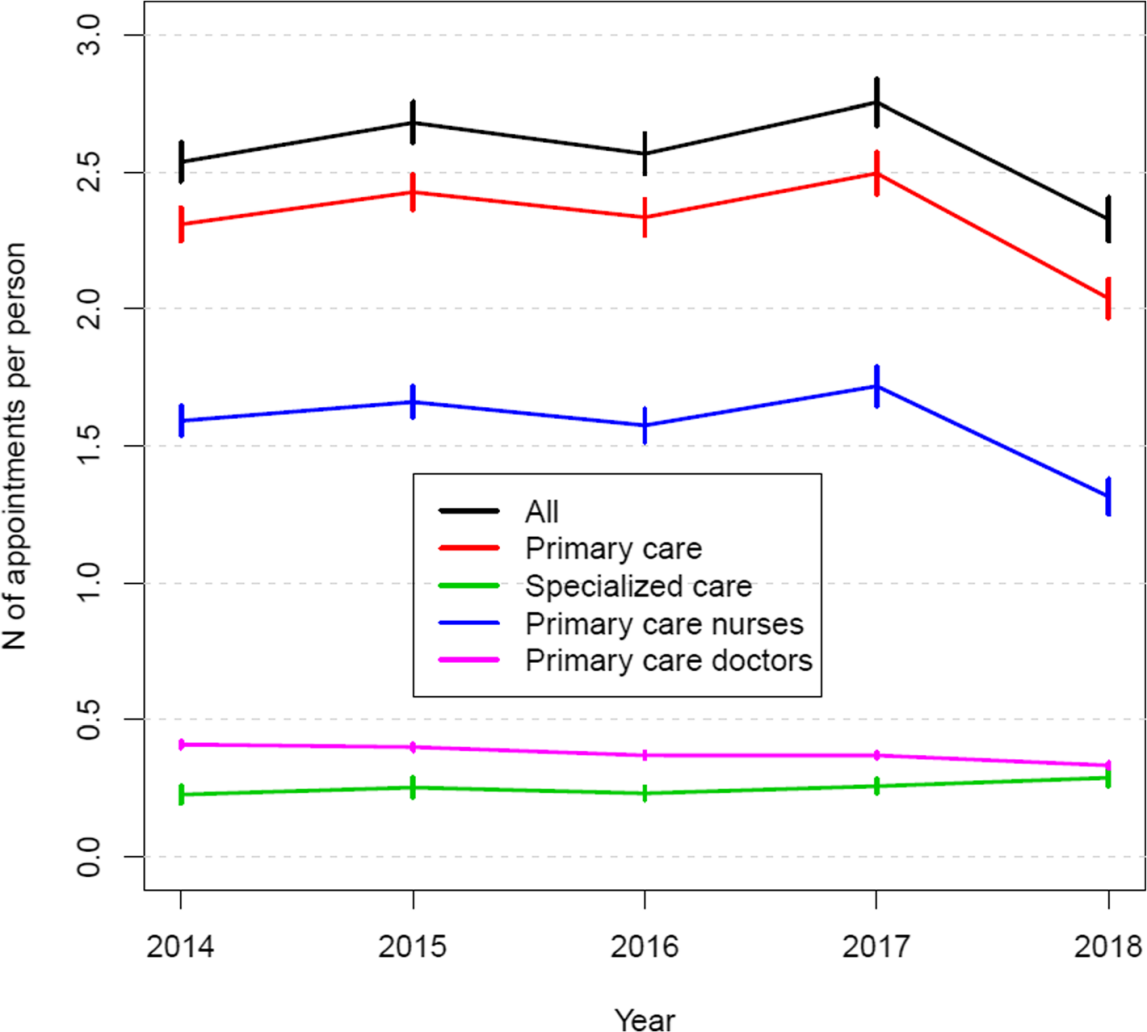
The mean number of appointments (with 95% CI) in primary and specialised health care

A similar and even stronger pattern can be seen in primary health care usage as seen in total health care usage (see Table 1). The mean number of contacts in primary health care was 2.99 in 2017 and 2.59 in 2018 (-0.40, *p* < 0.001), and the mean number of appointments was 2.49 and 2.04 (-0.45, *p* < 0.001) respectively (see Fig. 1). Between the years 2014 and 2018, the difference in the mean number of contacts was − 0.06 (*p* < 0.001), and in the mean number of appointments it was − 0.27 (*p* < 0.001).

In specialised care, the health care usage was higher after the establishment of the new organisation, being highest in 2018 with a mean of 0.29 contacts per person, a 0.06 increase from 2014 (*p* < 0.001). The proportion of specialised care contacts of all forms of contact was stable from 2014 to 2017 (at around 8%) but increased between the last two years by 2.1%p (*p* < 0.001), up to 10.1%.

### Health care usage by staff occupation

The difference in the mean number of contacts with primary care nurses was − 0.36 (from 2.18 to 1.82, *p* < 0.001) and for appointments it was − 0.41 (from 1.72 to 1.31, *p* < 0.001) between 2017 and 2018, the years after the establishment of Siun sote (see Table 2). For other staff groups, the changes from 2017 to 2018 were modest: a -0.02 change in the mean number of contacts for both primary care doctors (from 0.40 to 0.38, *p* = 0.007) and primary care dentists (from 0.41 to 0.39, *p* = 0.001). The corresponding change in the mean number of contacts was − 0.01 (from 0.18 to 0.17, *p* = 0.302) for specialised care doctors and 0.04 (from 0.08 to 0.12, *p* < 0.001) for specialised care nurses between the last two years.

**Table 2:** The number of users and contacts (appointments and phone calls*) by staff occupation

	Annual range	2014	2015	2016	2017	2018	*P*-value
**Nurses**							
* N* of appointments and phone calls*		23,403	25,622	25,798	29,002	25,073	
Proportion of users, %		60.9	62.7	60.4	63.2	59.9	< 0.001^a^
*N* of contacts per person, mean	0-168	1.99	2.14	2.05^a^	2.27	1.93	< 0.001^b^
*Primary care nurses*							
Proportion of users, %		60.4	62.2	59.9	62.6	58.9	< 0.001^a^
*N* of contacts per person, mean	0-167	1.92	2.06	1.97	2.18	1.82	< 0.001^b^
*N* of E11/T90-contacts per person, mean		1.50	1.51	1.39	1.45	1.15	< 0.001^b^
*N *of appointments per person, mean	0-166	1.59	1.66	1.57	1.72	1.31	< 0.001^b^
*N* of phone calls per person, mean	0–29	0.33	0.40	0.40	0.47	0.50	< 0.001^c^
*Specialised care nurses*							
Proportion of users, %		1.3	1.7	1.9	2.0	2.9	< 0.001^a^
*N* of contacts per person, mean	0-157	0.06	0.08	0.08	0.08	0.12	< 0.001^c^
**Doctors**							
*N* of appointments and phone calls*		6891	6962	6674	7388	7151	
Proportion of users, %		37.1	36.5	34.2	34.6	32.3	< 0.001^a^
*N* of contacts per person, mean	0–26	0.58	0.58	0.53	0.58	0.55	0.010^c^
*Primary care doctors*							
Proportion of users, %		32.5	31.6	29.9	30.3	28.0	< 0.001^a^
*N* of contacts per person, mean	0–13	0.42	0.41	0.38	0.40	0.38	< 0.001^d^
*N* of E11/T90-contacts per person, mean		0.31	0.30	0.27	0.26	0.24	< 0.001^d^
*N *of appointments per person, mean	0–13	0.41	0.40	0.37	0.37	0.33	< 0.001^d^
*N* of phone calls per person, mean	0–5	0.01	0.01	0.01	0.03	0.05	< 0.001^d^
*Specialised care doctors*							
Proportion of users, %		9.1	9.3	8.7	9.1	8.8	0.375^a^
*N* of contacts (= appointments) per person, mean	0–24	0.17	0.17	0.15	0.18	0.17	< 0.001^c^
**Dentist**^¤^ (99.6% from primary care)							
*N* of appointments and phone calls*		3615	4377	4895	5220	5066	
Proportion of users, %		14.7	17.2	17.7	18.1	17.6	< 0.001^a^
*N* of contacts per person, mean	0–22	0.31	0.37	0.39	0.41	0.39	< 0.001^c^

* Most of the phone calls come from primary care nurses (95%) and primary care doctors (5%). Only 9 phone calls came from the primary care dentist and 11 from specialised care nurses and none came from specialised care doctors or specialised care dentists

^¤^ Dentists’ contacts were mainly (99.6%) from primary care. The number of specialised care dentists’ contacts (= appointments) was just 86 during 2014–2018

^a^Logistic model, ^b^Linear model, ^c^Friedman test, ^d^Poisson model

Nearly all contacts with dentists were from primary care (99.6%); there were less than 30 contacts made with dentists per year in specialised care. Similarly, phone calls were mainly to primary care nurses (95%) and doctors (5%). The proportion of phone calls was 5% of all doctors’ contacts and 21% of all nurses’ contacts in primary care. More than two thirds of contacts with primary care doctors and primary care nurses had type 2 diabetes as the main reason for contact (ICD-10 = E11 or ICPC-2 = T90): 69.4% and 70.2%, respectively. In particular, the number of these contacts has decreased for primary health care nurses: change in the last two years in the mean number of contacts was − 0.30 (*p* < 0.001).

From 2014 to 2018, the differences in the mean number of all contacts and appointments with primary care nurses were − 0.10 (*p* < 0.001) and − 0.28 (*p* < 0.001) respectively. The corresponding numbers for contacts and appointments with primary care doctors were − 0.04 (*p* < 0.001) and − 0.08 (*p* < 0.001). The difference between 2014 and 2018 in the mean number of contacts with type 2 diabetes as a main reason (ICD-10 = E11 or ICPC-2 = T90) was much bigger than for all contacts: -0.35 for contacts with primary care nurses and − 0.07 for contacts with primary care doctors.

The mean number of contacts with specialised care doctors has been quite stable over the years (range: 0.03). The number of contacts with dentists (contacts mainly from primary care) and with specialised care nurses increased from 2014 to 2018; the difference in the mean number of contacts was 0.08 (*p* = 0.010) for dentists and 0.06 (*p* < 0.001) for specialised care nurses.

### The relation between care indicators and health care usage

The annual measurement activity of HbA1c was lowest in the year 2018 when 74.9% of patients were measured with the mean of 1.34 measurements per person (see Table 1). Correspondingly, the measurement activity was the highest in 2015: 77.0% of the patients were measured with the mean of 1.42 measurements per person. Those patients who were not measured were using less health care services: the maximum mean number of contacts was 1.21 in 2017 and the minimum was 1.09 in 2018. A similar pattern can be seen for those who were measured. The maximum mean number of contacts was 3.32 in 2017 and the minimum number was 2.92 in 2018 for those patients who were measured and within the target level of HbA1c. The corresponding numbers for patients who were measured but not within the target level of HbA1c were 5.06 in 2017 and 4.65 in 2018.

The difference in mean number of contacts between the last two years was similar for those who were measured: -0.40 (*p* < 0.001) for those who were within the target level of HbA1c and − 0.41 (*p* < 0.001) for those who were not within the target level of HbA1c. But the difference in mean number of contacts was just − 0.12 (*p* = 0.001) for those who were not measured. The difference in the mean number of contacts between the years 2014 and 2018 was − 0.05 (*p* = 0.011) for those who were not measured, -0.02 (*p* = 0.225) for those who were measured and within the target level of HbA1c and 0.12 (*p* = 0.001) for those measured and not within the target level.

## Discussion

The total number of health care contacts was highest in 2017, when Siun sote started to work, but it declined in the following year (2018) to the same mean number of contacts as in 2014 and to an even lower mean number of appointments than the number in 2014. In particular, this pattern can be seen in all contacts with primary care nurses and in contacts where diabetes was recorded as the main reason for making contact. This decreasing trend was visible during the last two years, and even visible before the establishment of the new organisation. The specialised care usage was quite stable over the years; only contacts with specialised care nurses slightly increased between the last two years. The decline in contacts with primary care might reflect the harmonisation and better targeting of services according to segmentation of patients. In addition, the simultaneous increase in contacts with specialised care nurses possibly indicates the better management of unmet needs. These findings are in line with the previous studies showing no significant effect on secondary-care utilisation, hospital use nor on the costs, but also, the opposite results exist [6, 8]. Actually, some previous studies have even shown unintended consequences, such as premature hospital discharges and the increased risk of readmissions [18].

Like the number of contacts, the number of HbA1c measurements and the proportion of patients with annual measurements decreased between the last two years. However, the proportion of those with good control did not decline. The decreasing proportion of measured patients may either indicate that patients are dropping out from the follow-up or that those who are within the target level of HbA1c are no longer followed yearly. The findings of this study showed that those who had made a smaller number of contacts and who were not measured had smaller decline in the number of contacts between the last two years compared with those who were measured. Those who were measured had a similar decline in the number of contacts among both those within the target level of HbA1c and those not within the target level, even though there was greater usage of health care services among those not within the target level of HbA1c. When comparing the years 2014 and 2018, the number of contacts only increased among those measured and not within the target level of HbA1c. At the same time as the integration, the organisation and the processes of diabetes care in North Karelia were restructured.

During years before Siun sote, each health centre had Diabetes Nurses (total number of 25 Diabetes Nurses, about 1 per approximately 500 diabetic patients) and specialized appointments for diabetic patients with injection drug therapy (e.g. insulin or GLP-1 agonist), and some of those 14 municipalities had also physicians with special competence in diabetes care or specialized in internal medicine. Furthermore, all primary care physicians and nurses took part in type 2 diabetes care in normal appointments. Though there already was some regional collaboration (e.g. regular meetings, regional education etc.), it was strengthened and unified when Siun sote was formed. Also, many care protocols were harmonized and regional knowledge of quality differences were adduced by collected EHR quality data and local research data. All this increased better awareness of current situation under single management, which formed the core of regional diabetic care model. It seems that these organizational changes and overall quality improvement efforts carried out for several years in the region by a network of professionals treating patients with diabetes have improved the treatment outcomes, reduced some unnecessary follow-up visits in primary care and targeted the contacts so they are more with those who are not controlled [19].

The main aims of the planned social welfare and health care reform in Finland, including the better integration of primary and specialised services, are to improve citizens’ well-being, to decrease socioeconomic differences in health and welfare, and to guarantee geographical equity and access to services. At the same time, there is a need to increase the productivity of services and to improve the quality of care and to restrain the increase in public health and social care expenditure [10, 20]. Some of the regions like North Karelia have executed the integration even though the national reform is still on hold. The integration of services has happed as a structural change, combining the responsibility for organising primary care and specialised care under the same service provider, the joint municipal authority for social and health care services. Based on these results, some improvements have happened in the rationalisation of primary care services, but at least so far, it seems that reform has been insufficient to implement integrated care to an extent that it would have had a significant effect on the utilisation of secondary-level care.

Effective systems change, in addition to macro-level change, would need simultaneous meso- and micro-level reforms. And those, on the other hand, need the planning of integrated services across an organisation, in different settings and at different levels of care, and obviously this takes some time to happen in a large organisation [21, 22]. Our data only included information on contacts for two years after the reform and thus cannot reflect the longer-term effects of the integration and possible meso- and micro-level system changes arising from the integration. One of the major barriers observed for health care integration and its effective implementation is that even the structural and financial integration performed therein is a failure to break down the service boundaries or give budget holders control over clients’ service use, which could explain why no effect is seen in secondary-care utilisation [23].

Cost savings in health care should occur through cost-effectiveness and quality improvement, which need comprehensive systems change [24]. Changing clinical practice in health service delivery is a complex task. Three critical contextual factors that determine the outcomes of change intervention have been identified [25]. These are organisational culture, leadership and implementation. Even if change is seeded through structural reorganisation, it rarely shifts established cultures and ways of working. The preliminary results from Siun sote show that the re-organisation of the service system first increased the contacts, and after that they decreased. The primary health care nurse appointments declined simultaneously with some increase in specialised care nurse contacts while good glucose levels were maintained. This might reflect that unnecessary control visits in primary health care are cut off and specialised know-how is provided for those in need. Furthermore, the early detection of type 2 diabetes has improved in the region [19], which might have caused increased need for secondary care consultations by finding more complex cases than previously. Previous studies have shown that nurse-led care should be an integral element of the health care services offered to type 2 diabetes patients, and nurse-led interventions have been shown to improve the treatment outcomes, such as the level of HbA1c [26–28]. However, the treatment of complex type 2 diabetes patients requires special skills [27]. The review by Baxter et al. [8] indicated that care integration may increase patient satisfaction and enable access to adequate services, but evidence for other outcomes – such as the number of clinician contacts, the number of general practitioner appointments and the cost – remains unclear [8].

From the beginning of the year 2011, the regional EHR system has covered all health centres in North Karelia as well as the central hospital [19]. EHRs have great potential for monitoring and improving the processes and quality of diabetes care [29, 30]. There is also some evidence that the introduction of EHRs supports communication between professionals and patients by e-mail and telephone, and thus reduces the demand for primary-care office visits [31]. This may partly explain the observed trends in the numbers of primary care appointments and phone calls during the whole follow-up period. Moreover, previous studies have indicated that telephone coaching for people with chronic conditions can be effective in improving health behaviour, self-efficacy and health status, especially among vulnerable populations who had difficulty accessing health services [32]. On the other hand, difficulties in linking different information systems have been brought up as one of the barriers in service integration [6]. In the case of Siun sote, operational information management and technologies have not been slowing down the implementation of the integration.

In the process of changing clinical practice in health service delivery, the successes and failures are greatly determined by the people and processes either enabling or resisting the change [33]. Successful change requires new roles and responsibilities, and willingness among healthcare professionals to co-work and co-learn [34, 35]. In Siun sote, the quality improvement of type 2 diabetes care has been active, and the quality of type 2 diabetes care at the primary-health care level has been shown to be very good [36], but there is always room for improvement.

### Strengths and limitations

The electronic health record system cover all type 2 diabetes patients living in the region of North Karelia and include data from both primary and specialized care. It minimizes selection bias, non-responsiveness of the patients and missing laboratory data. This study covers data from years 2014–2018 including only two years after the re-organization of health services. After longer follow-up time, it is possible to study the longer-term effects of integration on diabetes care, and also to analyze the cost implications of integration.

## Conclusions

The observed changes in health care usage and the outcomes of care after the integration of health services might reflect the better targeting and more concordant services in different service units in North Karelia, Finland. However, this short follow-up after the re-organisation does not prove either comprehensive systems change or seamless processes between primary and specialised care. The establishment of multiprofessional, seamless work practices requires planned procedures, the good coordination of daily work and the break down of service and budget boundaries. Therefore, the information from the following years will be of great interest as we will see if there are going to be remarkable cost savings and quality improvements in the care of patients with chronic conditions due to the integration of health services.

## Data Availability

The health records data analysed for the current study is not available in order to protect the privacy of the patients.

## Abbreviations

EHRs: Electronic health records
GEEs: Generalised estimating equations
HbA1c: Glycated haemoglobin
